# Biosensing with Paper-Based Miniaturized Printed Electrodes–A Modern Trend

**DOI:** 10.3390/bios6040051

**Published:** 2016-09-28

**Authors:** Célia M. Silveira, Tiago Monteiro, Maria Gabriela Almeida

**Affiliations:** 1 UCIBIO, REQUIMTE, Faculdade de Ciências e Tecnologia, Universidade NOVA de Lisboa, 2829-516 Monte de Caparica, Portugal; c.silveira@fct.unl.pt (C.M.S.); tc.monteiro@campus.fct.unl.pt (T.M.); 2 Instituto de Tecnologia Química e Biológica António Xavier, Universidade NOVA de Lisboa, Av. da República, 2780-157 Oeiras, Portugal; 3 Centro de Investigação Interdisciplinar Egas Moniz (CiiEM), Instituto Superior de Ciências da Saúde Egas Moniz, Campus Universitário, Quinta da Granja, 2829-511 Caparica, Portugal

**Keywords:** paper analytical device, printed electrodes, wax patterning, biosensors.

## Abstract

From the bench-mark work on microfluidics from the Whitesides’s group in 2007, paper technology has experienced significant growth, particularly regarding applications in biomedical research and clinical diagnostics. Besides the structural properties supporting microfluidics, other advantageous features of paper materials, including their versatility, disposability and low cost, show off the great potential for the development of advanced and eco-friendly analytical tools. Consequently, paper was quickly employed in the field of electrochemical sensors, being an ideal material for producing custom, tailored and miniaturized devices. Stencil-, inkjet-, or screen-printing are the preferential techniques for electrode manufacturing. Not surprisingly, we witnessed a rapid increase in the number of publications on paper based screen-printed sensors at the turn of the past decade. Among the sensing strategies, various biosensors, coupling electrochemical detectors with biomolecules, have been proposed. This work provides a critical review and a discussion on the future progress of paper technology in the context of miniaturized printed electrochemical biosensors.

## 1. Introduction

Over the last decades, there has been a significant emphasis on developing novel diagnosis systems that enable the analysis of complex samples on site and deliver fast but accurate results. Such demand for innovative and improved analytical methodologies comes mainly from the environmental monitoring, industrial control and health care fields. On the one hand, rules and directives restricting the level of many chemical and biological species in environmental samples, foodstuff and industrial products, are continuously being promulgated by several international agencies such as the European Union council (https://ec.europa.eu), the European Environment Agency (www.eea.europa.eu), the World Health Organization (www.who.int) and the Food and Agriculture Organization (www.fao.org) of the United Nations, as well as some National authorities, such as the Food and Drug Administration (www.fda.gov) and the Environmental Protection Agency (https://www3.epa.gov) in the United States. One of the major global health issues is the access to potable water in the developing world where its contamination, due to inadequate sanitation, is quite common; here, rapid tests are very helpful to check water safety and avoid waterborne diseases. In developed countries, the contamination of water supplies from industrial sources, human dwellings, agriculture practices, also have a strong impact on the ecological systems and ultimately, on public health, thereby requiring a strict monitoring [1]. Food safety is another major concern. The regulatory control of food additives, among other parameters, is mandatory to assure the quality of food products. Though, one of the main priorities is the timely detection of toxins and infectious agents in contaminated food, thereby protecting consumers from foodborne illnesses and other intoxications. On the other hand, clinical diagnosis is demanding optimal sensors, tailored for the specific needs of real-time analysis in physiological samples. Unquestionably, the quick determination of time-critical parameters such as blood metabolites and cardiac markers accelerates decision-making in emergency situations. This drives the development of point-of-care tests (POCT) i.e., devices that provide non-trained personnel with clinical results within a few minutes, either at home or healthcare services, thereby moving diagnostics from the laboratory to the bedside [1,2,3,4].

The need for effective POCTs is continuously increasing worldwide. This is driven not only by new discoveries in biomedicine and therapeutics (e.g., novel biomarkers, theranostics, personalized medicine), but also due to global trends such as the world demographic increase or the population ageing in developed regions [5,6]. Point-of-care diagnostics is also fundamental for the low- and middle-income countries due to the lack of infrastructural and financial resources, shortages in trained staff, the burden of endemic infectious diseases, and the increase in spurious and counterfeit drugs [1]. Although the stimulus for POCTs technology came clearly from the clinical diagnosis, the use of rapid and cheap tests at the point-of-need can also contribute to better environment protection and food safety, improving the living conditions of local communities in non-industrialized countries, especially those living in at-risk areas [1,4].

Therefore, substantial efforts have been made to produce analytical solutions of reduced costs, making the early diagnosis and detection of hazardous substances possible, even in the most resource-limited settings [5,7]. Because the lack of access to instrumentation maintenance and/or repair is also a constraint in developing regions, POCTs are preferentially designed to display readouts in small sensing devices or, ideally, in equipment-free systems. The World Health Organization outlined other key words in the research and development of POCTs for the lowest-resource environments. Altogether, they constitute the ASSURED guidelines: Affordable, Sensitive, Specific, User-friendly, Rapid and Robust, Equipment-free and Deliverable to end-users [1,4,7,8]. However, regarding the use of POCTs in the developed world, some of these criteria are not quite relevant. Instead, other sophisticated features such as multiplexing (simultaneously measure of multiple analytes) and connectivity to information systems (e.g., patient’s electronic health record) are more attractive [4,6,9]. Global markets are also looking for rapid diagnostic assays, operating with low-volume samples and eliminating needles and other sharps [1].

The recent advances in materials science, nanotechnology and (micro)electronics have been fundamental to miniaturizing the sensing devices, reducing the production costs, improving the analytical performance [10], and facilitating the inclusion of biological components for analyte detection, a crucial aspect in the construction of the so-called biosensors systems [9]. In this context, one of the most modern trends is the development of paper-based bioanalytical devices, such as the lateral flow strips made of (nitro)cellulose.

Paper offers several advantages, with its very low cost being the most highlighted benefit in the literature. In fact, this naturally abundant and renewable material can be machined using similar methods to those traditionally employed, for example, in microfluidic-based diagnostics; yet, it is substantially cheaper and easily adaptable to mass manufacturing processes. Another asset relates to the facility on discarding this eco-friendly material by incineration, thereby avoiding hazardous waste. Because fluids can move throughout the porous substrate by capillary action, there is no need for pumping systems and only small sample volumes are required. Moreover, patterning paper with hydrophilic channels delimited by hydrophobic walls allows samples to be dispensed into several spatially separated zones, which enables running multiple assays simultaneously, on a single device. All these characteristics are highly valuable for POCTs tailored not only for resource-limited settings but also for applications in developing regions, opening up a world of new applications for an older player, as paper is [1,6,8,11,12].

Besides the lateral flow assays mentioned before, paper-sensing platforms have currently been utilized in other configurations, including dipstick tests and microfluidic paper-based analytical devices (μPADs). The latter were proposed in 2007, in a landmark work by Whitesides et al. [13] that attracted much attention in the lab-on-a-chip community and challenged many other researchers to create innovative products. As such, the development of POCT devices using cellulose-derived matrices is currently an area of active research and has been a matter of recent reviews, some of which have been cited throughout this manuscript.

Herein, a strong focus will be given to paper-based biosensors integrating miniaturized printed electrodes for signal transduction. Other interesting works that do not report simultaneously biosensing and electrochemical detection, on paper based printed platforms, were considered out of the scope of the present manuscript. Readers interested in additional topics not covered in this work (paper sensors, colorimetric detection, etc.) are referred to recent review articles in this field [12,14,15,16,17,18].

The paper electrochemical biosensors trend is an added value to the diagnostic assays since it combines the merits of paper as a solid support, to the high sensitivity and selectivity of bioelectrochemical detection. In fact, while the functionalization of the working electrode with a biological component markedly improves analyte recognition, the electrochemical transducer provides quantitative readouts, with good detection limits, especially if nanostructured materials such as metal nanoparticles, carbon nanotubes or graphene nanosheets are used for signal amplification [12]. Moreover, the electroanalytical techniques are less prone to interferences than other quantitative methods (e.g., photometry, chemiluminescence, electroconductivity, fluorescence), avoid sample pretreatment and are easier to miniaturize [6,19,20]. Consequently, bioelectroanalytic systems have become the method of choice for point-of-care analysis, being well established in the market [9,20].

Technically, the electrochemical cell is composed of a three-electrode system, including a reference, a counter and a working electrode. The electrodes are made of conducting inks (carbon or metal) using mass production techniques, such as screen-printing, inkjet-printing or pencil-drawing [12]. The working electrode is the key component, because it is the place where the recognition event is transduced. Typically, the electrode surface is coated with an enzyme extract (whether or not purified) that catalyzes an electron transfer reaction in the presence of its substrate, the analyte. Depending on the species that interacts with the working electrode, namely a reaction’s product/co-substrate, a redox mediator (mediated electrochemistry) or the enzyme itself (direct electron transfer), one can have first, second or third generation biosensors, respectively. In all cases, the generated current is directly correlated to the analyte concentration [21]. Nevertheless, other types of biological components such as antibodies, nucleic acids, and receptors, can be coupled to electrochemical surfaces; because the analyte detection results from a ligand-receptor reaction, these analytical devices are classified as affinity biosensors [9].

In general, the immobilization of chemical (e.g., redox mediators, nanostructured particles) or biological molecules (e.g., proteins, DNA) is a critical step for (bio)sensors functioning. However, these species can be easily immobilized on a paper substrate by just drop casting a solution containing the required compounds (solubilized or suspended), followed by solvent evaporation [11,12], or through sophisticated methodologies that now exist and allow the highly controlled deposition of functional molecules on solid substrates. In particular, screen-printing and, specially, inkjet-printing technologies have the capacity of depositing small droplets (pL) of a liquid solution (e.g., conductive ink, protein containing sample) on paper, with high precision and resolution, according to a previously defined pattern [22]. Furthermore, paper can work as the immobilization matrix of the (bio)recognition components; in this simple approach, a small disk of modified paper is placed in direct contact with the working electrode surface.

To conclude, the association of bioelectroanalytical techniques with miniaturized paper-based platforms holds great promise in the field of point-of-need tests, for all markets (environmental, food and clinical analysis) and for both the developed and developing worlds. The devices are portable, easy-to-use, with rapid turn-around, affordable, and are based on a self-sustainable and biodegradable material. This review paper addresses the current state-of-the-art, in this still recent trend.

## 2. Paper-based Electrochemical Biosensors

### 2.1. Paper Substrate

As the central component in paper-based electrochemical biosensors, paper is not only the support substrate for the electrodes but also the matrix where sample and recognition biomolecules are joined and react. In fact, paper offers a thin, mechanically stabilized film of water, or other fluids, that deliver analytes to the surface of the electrodes [23]. It is also the regulator of the fluidic properties and the geometry of the device. In addition, the three-dimensional (3D) network of cellulose fibers is the base for deposition of redox and conductive materials, which can augment the surface area and conductivity of the resulting devices.

The majority of publications on paper-based electrochemical biosensing platforms (Table 1) resorted to Whatman grade 1 filter [24,25,43] or chromatography paper [23,30,44]. The fibers in Whatman paper originate from high quality cotton linters, which are treated in order to achieve a high alpha-cellulose content (>98%) and to guarantee quality, reproducibility and uniformity [45]. The high water absorbency and biocompatibility of this type of paper is well suited for the construction of bioelectrodes. Exceptions to Whatman grade 1 paper were proposed by Noiphung et al., which employed Whatman VF paper in combination with the former and by Määttänen et al., which made use of multilayer-coated recyclable paper [28,29].

### 2.2. Hydrophobic Pattern Design

As mentioned earlier, due to its porous nature and surface chemistry, paper is a very hydrophilic matrix [18,45]. Aqueous solutions can easily travel through the cellulose 3D network spaces by capillarity and viscous solutions can also diffuse vertically. In order to define specific regions for electrode printing and reaction zones, hydrophobic patterns must first be defined in the paper substrate. To this end, the porous network of the cellulose matrix is filled with hydrophobic materials, such as wax, photoresist or hydrophobic polymers, to produce barriers that delimit the hydrophilic microchannels.

The most common hydrophobic patterning method is wax printing (cf. Table 1), where commercial wax printers are used to transfer the designed pattern onto a paper sheet, which is then baked, allowing the melted wax material to vertically diffuse through the porous cellulose fibers network [17,30,31,43]. Alternatively, other methods such as wax dipping, photolithography, plasma treatment and inkjet-printing with polydimethylsiloxane (PDMS) or alkenyl ketene dimer based inks have been reported [23,24,28,29,45,46,47].

### 2.3. Electrode Set-up

**Figure 1:**
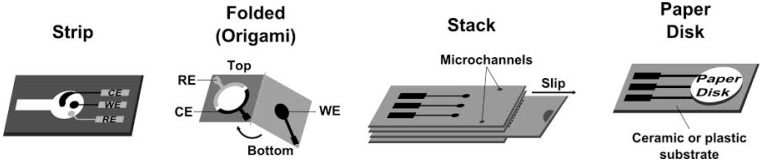
Configurations of paper analytical devices used for electrochemical biosensing. RE–reference electrode, CE–counter electrode, WE–working electrode. Schemes were adapted from the following references: strip [24], folded [34], stack [40], paper disk [32].

Paper-based electrodes can be easily produced in various configurations (Figure 1) due to the inherent properties of this material, including thickness, flexibility and hydrophilicity. Consequently, paper-based platforms can be tailored to any desired application. In the context of electrochemical biosensors, paper materials have been used as a support substrate for all the electrodes composing the electrochemical cell (working, reference and counter) [24,31,35], but also in combination with conventional electrodes [25,26].

In the most basic approaches, the devices are prepared in single paper pieces where the electrodes are deposited, generally on circular or channel-like hydrophilic test zones [35,42]. Multiple electrodes have also been fabricated in the same strip and used for multi-analyte detection (array sensors) [24,31,33]. In addition, single paper pieces were used as biomolecule immobilization layers and liquid transport or separation pathways (cf. paper disk set-up in Figure 1) [26,29].

Several proposals were presented with origami configurations; in these cases, the paper was folded to create a 3D device [34,43]. Usually, the working electrodes were prepared in one segment of the paper, and the counter and reference electrodes fabricated in another. Paper-based biosensors have also been produced in different sheets of paper that were stacked together for operation [36,40]. In either configuration, the multilayers of paper improve the wettability of the electrodes, which can enhance the analytical performance of the devices. Moreover, the additional sheets of paper may be used to separate and filter components in the sample [30,40]. The multi-layered devices have also been applied in dual phase procedures; for example, in some origami assemblies, the working electrode segment was pre-incubated with the sample and afterwards the device was folded to perform the electrochemical measurement [36].

### 2.4. Electrode Construction Methods

Different methods have been used for fabricating the electrodes in paper-based electrochemical biosensors. We have grouped the proposals according to the type of electrodes, namely screen-printed (and related stencil-printed), inkjet-printed, graphite-pencil based and commercial electrodes coupled with paper materials. In the following sections we will discuss their characteristics and operating principles.

#### 2.4.1. Screen-printing

In the majority of works, the electrodes (working, counter and reference) are screen-printed on the hydrophilic areas of single strips or multiple pieces of the paper substrate, according to a defined pattern. In both cases, the electrochemical cell is contained within the paper device in a configuration ready to be used outside the lab. However, other proposals still make use of conventional auxiliary electrodes and electrochemical cells in combination with a working screen-printed electrode (SPE) which is dipped into the sample solution [25].

Carbon derived inks or pastes are generally used for printing the working and counter electrodes, whereas Ag/AgCl materials are applied as reference electrodes. Carbon pseudo-reference electrodes have also been described; in this case the sensor fabrication process is facilitated because the electrodes can be prepared in a single screen-printing step [23,31,42]. In some devices, Ag materials are also used as a base layer for improving electrode contacts.

Electrode modification with biomolecules, polymers or nanostructured materials has been achieved through various techniques. The most recurrent and simple one is drop casting; however, other methods, such as electrodeposition, chemically induced polymerization and self-assembling, have also been used. This subject will be discussed below for several paper-based electrochemical biosensors prepared by screen-printing techniques.

In respect to the biorecognition elements, these are usually oxidase enzymes (e.g., glucose oxidase/GOx, cholesterol oxidase/ChOx). In most cases, their catalytic activity towards the target molecule can be monitored indirectly through the reduction of redox mediators, according to a second generation biosensor transducing scheme. Nevertheless, the direct measurement of an electroactive product of the reaction (e.g., H_2_O_2_) in first generation biosensor configurations [35], or direct electron transfer based enzyme biosensors (third generation) were reported [32,43]. Several screen-printed paper-based affinity biosensors have also been proposed (e.g., immunosensors) [34,38]; although the biorecognition event is based on a non-catalytic process (e.g., antigen–antibody interaction) these are frequently coupled with signal amplification systems composed of redox enzymes or enzyme-like electrocatalysts.

##### (a) Enzyme Based Catalytic Biosensors

**Figure 2:**
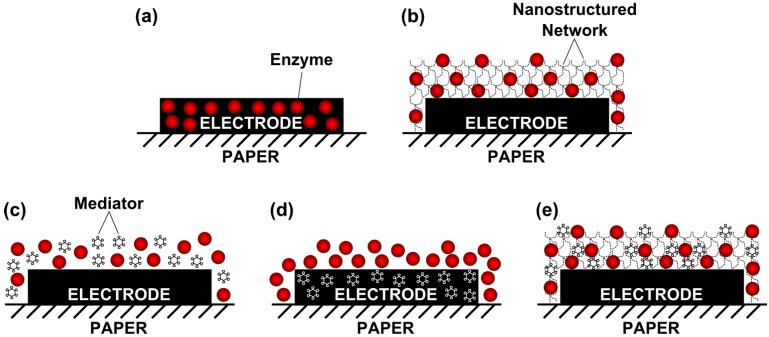
Surface modification strategies of paper-based enzymatic biosensors: (**a**) inkjet-printed graphene–enzyme composite; (**b**) biomolecule drop cast over nanostructured layer; (**c**) biomolecule and redox mediator mixture drop casting; (**d**) screen-printed carbon–redox mediator composite with biomolecule drop casting; (**e**) biomolecule and redox mediator mixture drop casting over nanostructured layer.

A straightforward method for the preparation of screen-printed paper-based biosensors consists of casting the enzyme solution or enzyme/redox mediator mixture on the paper test zones (where the electrodes are printed) and allowing it to dry (Figure 2c). The materials are absorbed on the paper substrate and available to react with the target molecule upon contact with the sample solution. Nie et al. developed a microfluidic paper-based glucose biosensor using this strategy [23]. The enzyme (GOx) was applied by drop casting on the paper microchannels in a mixture with ferricyanide as redox mediator. The electrode strip was overlaid by an additional paper channel assembled on top of the electrodes, which acted as a sink to maintain the fluids continuously on the electrodes. The biosensor was used to quantify glucose in artificial urine by amperometry. The sensitivity of this paper-based biosensor was only slightly lower than that of a similar carbon bioelectrode used to measure glucose in bulk solution, whereas the detection limit (0.22 mM) was below the value attained from commercial glucometers and reported for colorimetric paper-based platforms [23].

A very similar approach was used for the preparation of screen-printed biosensor arrays for the simultaneous detection of glucose, lactate and uric acid [24,31]. The preparation of the biosensing layers was simply achieved by drop and drying small droplets of enzyme containing buffer solutions (GOx, lactate oxidase or uricase) on their respective individual electrode modules. Zhao et al. described a biosensor array comprising of eight individual sets of electrodes, which were modified with potassium ferricyanide deposited with the enzyme mixture [31]. The current responses to the analytes were measured by amperometry on artificial urine, spiked with different concentrations of glucose, lactate or uric acid. The biosensor array was coupled with a handheld, custom-made potentiostat which enabled multiplexed readings. The analytical performance of the device was comparable to that of commercial meters and other paper-based devices [31]. In the case of the study by Dungchai et al. (the first report of a paper microfluidic device with electrochemical detection) the working and counter electrodes were impregnated with the redox mediator (Prussian Blue) which was mixed with the carbon ink before the screen-printing process (Figure 2d) [24]. In this way, only the enzymes were drop-cast on the working areas of the proposed triple-analyte biosensor. The bioelectrodes were tested by amperometry and yielded low limits of detection, within the ranges found in biological samples (e.g., serum, blood, and urine) in the case of glucose and lactate. This device was also applied in the analysis of human plasma samples, proving to be efficient for the detection of the target analytes [24]. In a related work, Dungchai and co-workers combined the above described glucose biosensing platform with a paper-based colorimetric assay for total iron content determination in human serum [48].

Aiming to improve the performance of paper-based biosensors, some authors have employed additional electrode modification materials, such as conducting polymers and nanoparticles (NPs), aside from the biomolecules and redox mediators. This trend was followed in the construction of a cholesterol biosensor, based on ChOx as a sensing element [35]. The enzyme was applied by drop cast over a layer of graphene, polyvinylpyrrolidone and polyaniline (PANI) nanocomposite, previously electrosprayed onto the screen-printed carbon electrode (SPCE) surface (Figure 2b). This generated a droplet-like nanostructure with a high surface area that allowed a significant enhancement of biosensor sensitivity. Contrary to the previous works using redox mediators, the activity of the bioelectrode was measured amperometrically via the direct oxidation of the H_2_O_2_ produced from the ChOx enzymatic reaction (first generation biosensor). The bioelectrode was proposed as a simple and inexpensive method for cholesterol quantification, since the comparison with other electrochemical biosensors showed that the analytical performance (sensitivity, limits of detection and dynamic ranges) was either improved or equivalent to the previous works. The sensor was further tested in spiked human plasma samples providing a high accuracy [35].

In a work by Wu et al. towards the development of an ethanol biosensor, the electrodes were spotted with 3-aminopropyldimethylsiloxane in order to improve wettability and amplify the electrochemical signal [42]. The detection mixture containing the enzyme (alcohol dehydrogenase, ADH), the nicotinamide adenine dinucleotide (NAD^+^) cofactor and the redox mediator (potassium ferricyanide) was then drop casted and dried on the surface of the device’s test area (Figure 2e). Interestingly, when the enzyme/mediator solution was directly mixed with the sample just prior to measurement, the freshly deposited reagents provided superior results. Therefore, the authors added an additional modification material to the biosensor, trehalose, which acted as an enzyme stabilizing agent, allowing the biosensor to maintain its initial activity after 48 h of storage at 4 °C. The electrode response to ethanol was measured by amperometry. The paper biosensor was adapted for use with a commercial glucometer, therefore turning this device into a simple miniaturized low-cost platform for the measurement of ethanol [42].

As aforementioned, a key point for the application of any type of electrochemical biosensor outside lab settings is the implementation of portable, simple-to-use signal readout instruments. In this context, Fischer and co-workers developed a self-powered glucose monitoring system based on an enzymatic fuel cell design composed of paper electrodes as cathode and anode [43]. The device did not require any external power sources, since it used the energy produced by the reactions occurring at the fuel cell electrodes. The cell was constructed as a 3D origami structure, with three sections of paper folded over each other: the anode layer, the anode reservoir, and the aircathode layer. The anode constituted the biosensing part of the device; it was modified by drop casting with a mixture of GOx and chitosan (Figure 2b). This material was used to increase the specific surface area of the electrode, thereby improving the enzymatic direct electron transfer reaction and the electrode sensitivity. The electrons generated by the catalytic reaction on the anode were directed towards the cathode (where the paired reduction reaction occurred) and then through the external circuit, therefore powering the device. A simple digital multi-meter could be used to monitor the glucose added to the cell, since the potential difference measured between the two electrodes (converted to current) was proportional to glucose concentration, within the range 1–5 mM. The device had a high internal resistance, probably due to poor direct electron transfer in the bioanode. Nevertheless, it enabled stable current generation, provided enough glucose was added to the reservoir [43].

##### (b) Affinity Biosensors

Affinity biosensors are based on very selective binding interactions between the target analyte (ligand) and a biological component such as an antibody, nucleic acid, or a receptor [49]. The paper support is a promising platform for the development of these sensors, since the low-cost material may cut-down the frequently high production expenses (e.g., antibodies).

A common feature in most screen-printed paper-based affinity biosensors is the use of highly conductive materials and/or nanostructured constructions. This is done with the aim of increasing the load of adsorbed biomolecules (and consequently ligand/receptor complexes) and therefore improve the sensitivity. Additionally, the preparation procedures and assays of these sensors typically involve multiple immobilization/incubation steps.

A simple point-of-care diagnostics immunosensor for troponin (a cardiovascular injury biomarker) was developed on a screen-printed conducting paper [25]. The device was implemented as a three electrode electrochemical cell composed of the working electrode and conventional Pt and Ag/AgCl electrodes as counter and reference, respectively. The working interface was modified with PANI using an electrochemical deposition method. This approach enabled the creation of a stable conducting polymer coating on the paper (PANI nanostructure). Anti-cardiac Troponin-I (Anti-cTnI) was immobilized on the PANI SPCE through cross-linking. The biosensor response was monitored by cyclic voltammetry; the change in the oxidation current of PANI was proportional to the concentration of troponin added to the electrode. According to the authors, the formation of the Anti-cTnI/troponin affinity complex on the surface of the electrode created an electron-transfer-accelerating layer with an excess of positively charged residues (from troponin). Therefore, the negatively charged redox mediator, ferricyanide, was attracted towards the interface facilitating the redox reaction. The current detected varied linearly with the concentration of troponin within a wide physiological range (1–100 ng/mL) and with high sensitivity. The immunosensor displayed good reproducibility upon several consecutive experiments [25].

**Figure 3:**
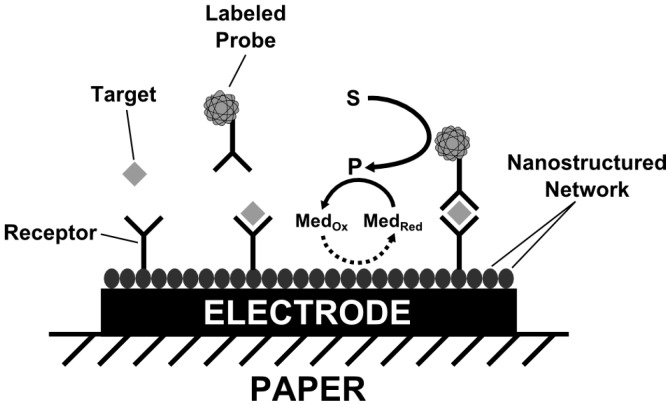
Working principle of affinity biosensors based on sandwich-type assays coupled with electrochemical detection. S–enzymatic substrate, P–product, Med–redox mediator.

A main concern regarding amperometric affinity biosensors is the relatively small current variations associated with ligand-receptor reactions. Therefore, signal amplification strategies are crucial to improve the analytical performance [50]. The following affinity sensors all couple the ligand-receptor binding assay with electrochemical detection systems using enzymes or nanoparticles with enzyme-like activities for signal amplification. The working principle of these devices is based on sandwich-type assays, where, after binding the target molecule to the electrode immobilized receptor, a secondary catalyst-labeled probe is added. Thereafter, the catalytic reaction is triggered by the addition of an enzymatic substrate and the resulting current signal is related to the amount of receptor-ligand complexes (Figure 3).

Li et al. used this strategy to develop an immunosensor for the detection of prostate protein antigen (PSA), which relied on the redox cycling of GOx for signal generation [34]. The device was constructed on a 3D origami paper support composed of two sections (auxiliary and sample) which were folded over each other to create the electrochemical cell. A 3D structure with a high surface area was generated on the sample segment by sequential growth of gold nanoparticles (AuNPs) and electrodeposition of MnO_2_ nanowires. The capture anti-PSA antibody (McAb1) was then absorbed onto the AuNPs. After incubation with PSA, carbon nanosphere labels loaded with GOx and signal anti-PSA antibody (McAb2) were incubated with the device and attached to the immobilized PSA. The amount of bound PSA was measured via the GOx enzymatic reaction. The current response (measured by differential pulse voltammetry, DPV) in the presence of glucose and 3,3',5,5'-tetramethylbenzidine (TMB) as redox mediator, was directly correlated with the concentration of PSA. The electrodes had a low detection limit and high sensitivity, which was enhanced by the porous morphology and high surface area of the electrode construction. The biosensor presented good accuracy, compared to the reference method, when used in human serum samples [34].

A similar approach was proposed by Ge and co-workers for the construction of a paper-based biosensor for detecting K-562 cancer cells [38]. For this purpose, concanavalin A (Con A) was immobilized in a highly conductive and biocompatible electrode surface prepared with composites of 3D-AuNPs/graphene oxide (GN) and ionic liquid (IL) films (1-ethyl-3-methyl imidazolium tetra-fluoroborate ([BMIM]BF4)). As in the previous work, auxiliary and sample areas with the SPEs were folded over each other to assemble the device. The Con A-modified IL/3D-AuNPs/GN electrode was used for capturing K-562 cells. The electrochemical detection of K-562 cells was based on a sandwich-type assay using paladium-silver nanoparticles (PdAg NPs) labeled with Con A, as peroxidase-like catalysts for the reduction of H_2_O_2_ (released after cell stimulation with phorbol 12-myristate-13-acetate, PMA) and thionine as electron mediator. The resulting redox reaction was monitored by DPV. The bioelectrode had good reproducibility, was highly sensitive for the determination of the H_2_O_2_ and presented a low detection limit [38].

The same authors developed another K-562 cells biosensor on a related origami paper-based platform [39]. The cells were captured by folic acid-functionalized AuNPs grown on the surface of the cellulose fibers, in the paper sample zone of the SPCE. The NPs improved the conductivity and increased the effective surface area of the bare working electrode. The resulting sensing interface could selectively bind to the folate receptors over-expressed in the K-562 cells. Hybrid gold–paladium–platinum (Au@PdPt) NPs with intrinsic peroxidase-like catalytic activity were used for signal detection. The Au@PdPt nanoprobes (functionalized with folic acid for cell recognition) catalyzed the oxidation of thionine by H_2_O_2_ added to the sample zone. The electrochemical response was monitored by DPV. As previously observed, the high electron conductivity and specific area of the nanostructured interface contributed to the good performance of the biosensor. Additionally, the stability of the catalytic nanoprobes was improved in comparison with redox enzyme based assays (e.g., horseradish peroxidase, HRP). The sensor also demonstrated good precision and reproducibility with relative standard deviations of intra-assays and inter-assays lower than 5% [39].

A multiplexed electrochemical platform of paper based immunosensors was developed by Wu and co-workers for the detection of cancer biomarkers, namely carcinoembryonic antigen (CEA), alphafetoprotein (AFP), cancer antigen 125 (CA125) and carbohydrate antigen 153 (CA153) [36]. The device was fabricated as two square pieces of paper overlaid together, the first having a central circular zone connected to eight surrounding test areas with SPCEs and a second matching paper piece containing shared carbon counter and Ag/AgCl reference electrodes. The capture antibodies for CEA, AFP, CA125 and CA153 were drop casted over layers of graphene oxide and chitosan, in the presence of glutaraldehyde. As in the previous proposals, after the capture of target molecules on the immunodevice surface, an enzymatic electrochemical detection system was added in order to amplify the signal. In particular, polymeric chains of glycidyl methacrylate (GMA) where generated, via a controlled radical polymerization reaction, on the surface of the device for the binding of HRP. Upon addition of the enzyme’s substrate, H_2_O_2_, and the redox mediator O-phenylenediamine, a current signal (directly correlated with the concentration of cancer biomarker) was measured by DPV. The concentration ranges obtained were appropriate for measuring the levels of cancer biomarkers that occur in human blood, plasma or serum and the limits of detection were improved in comparison with previously proposed methodologies. Additionally, the cross-reactivity between the different antigens and antibody modified electrodes was negligible, indicating that the device could be used for multianalyte detection. The response of the paper based immunodevices was maintained up to three-weeks of storage, indicating a good stability [36].

A different type of paper-based affinity sensor was developed by Li and co-workers for the detection of a 30 nucleotide long sequence of the hepatitis B virus [40]. The assay included a DNA hybridization reaction before the paper based electrochemical analysis. In the reaction pre-step, the target DNA was mixed with magnetic microbeads (MμBs) functionalized with the capture DNA and silver nanoparticles (AgNPs) modified with label DNA. The resulting three-strand DNA sandwich was then applied onto the paper device, which housed three carbon electrodes fabricated by stencil-printing. The oxidation of the AgNP labels by MnO_4_^–^ (previously added to the sensor) yielded soluble Ag^+^ that could be electrodeposited on the working electrode and detected by anodic strip voltammetry. The paper test combined a simple origami assembly with microfluidic hollow channels to accommodate micrometer scale particles. Additionally, a slip layer was used to control the timing of incubation steps whereas a magnet allowed concentrating the MμB-conjugated DNA sandwich on the surface of the working electrode. The combination of the MμBs and AgNPs provided signal amplification over 250,000 fold and a detection limit of 85 pM [40].

#### 2.4.2. Inkjet-printing

Along with other printing methods, inkjet-printing has become one of the most promising techniques capable of manufacturing cost-effective, disposable biosensors. It provides significant advantages over other related fabrication technologies, such as screen-printing, because of the versatility, the high precision and resolution of the printed patterns and importantly the ability to deposit very small volumes of ink and/or biomolecules (picolitres) in a rapid and low-cost procedure [51]. Although it is expected to grow in importance in the field of electrochemical paper sensing devices based on biological recognition, so far it has only been applied in a couple of works.

The hydrophobic pattern materials and respective methods of preparation, as well as the nature of the electrode conductive inks of paper-based inkjet-printed biosensors, are identical to those previously described for screen-printed bioelectrodes. Similar interface modification strategies have also been applied. For example, in the glucose biosensor proposed by Määttänen et al., the enzyme (GOx) was entrapped in a poly-3,4-ethylenedioxythiophene (PEDOT) film electropolymerized on the working electrode’s surface [28]. The biosensor was developed on an inkjet-printed platform based on multilayer-coated recyclable paper. The electrodes consisted of printed colloidal gold (working and counter) and Ag (reference) strips, the latter being covered with an additional layer of electrochemically deposited Ag/AgCl. The low-cost device could perform selective glucose sensing by amperometry with a linear response range from 0.1 up to 5 mM [28].

In addition to the common electrode modification methodologies used in biosensors, inkjet-printing enables a simplified procedure of simultaneous electrode preparation and incorporation of the biological component. Labroo et al. demonstrated this strategy in the development of graphene-based electrical biosensor arrays for the multiplexed detection of glucose, lactate, xanthine and cholesterol [33]. Microfluidic channels were created on the paper substrate in a cross pattern, producing four specific sample flow channels. At the end of each channel, a specific enzyme-graphene ink combination was printed, with either GOx, lactate oxidase, xanthine oxidase (XO) or ChOx (Figure 2a), to form each graphene biosensing electrode. Finally, highly conductive silver paste was printed (positive and negative terminals) so that the graphene electrodes could be connected to the potentiostat. The samples were added on the inlet, flowing to the different electrodes at the end of each channel. The analytes (glucose, lactate, xanthine or cholesterol) were oxidized by the corresponding enzyme and local H_2_O_2_ concentration increased, producing an increment in the measured oxidation current. The device performed rapid (less than 2 min) and simultaneous measurements with linear ranges between 0.3 and 15 µM. Additionally, it was shown to be accurate for measuring multiple metabolites in real blood samples [33].

#### 2.4.3. Graphite-pencil Based Working Electrodes

Common household graphite pencils represent a versatile but simple and low-cost alternative source for the fabrication of working electrodes, that has been recently rediscovered and applied in the field of electrochemical (bio)sensing paper devices. In contrast with screen- and inkjet-printing, pencil-drawing does not require supporting equipment, which may further lower the costs of biosensor fabrication and contribute to an easier implementation in resource limited areas. Pencil-drawing is simple, rapid and enables the construction of customized designs and geometries of prototypes for paper analytical devices. Furthermore, it is a solvent free process, since it does not resort to organic solvents commonly used in carbon pastes.

As an example, Li et al. were able to draw a complete three-electrode system with a commercial 6B grade pencil (Staedtler Mars, Germany) for the detection of glucose on a folded (origami) piece of paper [44]. The analytical device was comprised of two distinct areas—a circular hydrophilic delimited reaction zone where GOx was immobilized by drop casting, and an electrochemical detection zone, with fully drawn pencil electrodes and ferrocene carboxylic acid (redox mediator) also deposited by drop cast. The two areas were physically separated by a central crease that, when folded, allowed for the operation zones to come into contact and samples to flow through the reaction layer and into the detection area. The paper device provided linear current response to glucose up to 12 mM (measured by amperometry) and glucose readings in four human blood samples were comparable to those obtained with a commercial blood glucometer. Furthermore, the device had a good performance when tested for interference from species normally coexisting with glucose in physiological samples (e.g., dopamine, ascorbic and uric acid) [44].

Alternatively, Santhiago et al. used graphite pencils type H (Pentel, America) inserted in a glass capillary to manufacture the working electrode used on another folded paper-based glucose biosensor [30]. The device had three separate wax patterned areas of operation: (a) sample filtration; (b) enzymatic reaction; (c) electrochemical detection. GOx and the redox mediator, 4-aminophenylboronic acid, were spotted within the hydrophilic microzone in region (b) and silver ink was used to construct the reference electrode in region (c). Prior to glucose measurements, the paper device was folded, overlapping the three regions, and a drop of artificial blood serum sample or standard solution was added to region (a), flowing through region (b) and being collect at region (c), all in 4 min. Afterwards, the device was unfolded and the graphite pencil working electrode was positioned vertically and lightly touching the paper surface of region (c). Glucose was detected in the concentration range 0.01–1.5 mM, by measuring the oxidation of the redox mediator amperometrically. The device was able to quantify glucose in spiked artificial blood serum samples with recovery values of around 100% [30].

#### 2.4.4. Commercial Electrodes Coupled with Paper Based Analytical Devices

A more conventional approach that also takes advantage of paper characteristics consists of coupling commercially available electrodes (mostly SPCEs) with a small piece of paper, that usually serves to retain the biological recognition element. The strategy is practical and simple as the cellulose matrix can be used as an immobilization layer in direct contact with the electrode surface. Some examples are given below.

Several authors have proposed glucose biosensors based on commercial SPCEs overlaid by paper disks or pads previously impregnated with either GOx, GOx and redox mediator mixtures or the glucose sample to be analyzed (cf. Figure 1) [11,26,32,37]. In addition to the paper, polymeric or conductive material layers may also coat the working electrodes surfaces. For example, Kong et al. modified the working interface by drop casting a composite of graphene, PANI and AuNPs. This film improved the adsorption of GOx and promoted a favorable microenvironment for direct electron transfer between the enzyme and the electrode [32]. Similarly, Yang and co-workers functionalized SPCEs with AgNPs. The authors also stabilized the electrode-paper pad assembly by clamping it with acrylonitrile butadiene styrene plastic holders that held the paper layer in place during testing [37]. Tan et al. improved the paper disk concept by coupling a flow system (PDMS chip and shim on top of the paper disk and SPE) integrated with a sequentially reagent-loaded disposable cartridge, made of 200 cm long silicone tubing and secured with clamps in each terminal [26]. Noiphung et al. followed a different strategy than the previous authors and constructed a paper-based microfluidic device that used a combination of two paper separation zones (made of VF type paper) and a central detection region (grade 1 filter paper) placed on top of a commercial Prussian Blue SPCE [29]. Dumbbell pattern designs, prepared by wax dipping in the different papers’ sections, enabled homogenous whole blood separation and generated a uniform plasma flow to the electrode surface. Therefore, the reproducibility of the electrochemical signals was greatly improved. Furthermore, the wax dipping process helped to physically attach the paper separation and detection zones. As in the previous works, GOx was immobilized on the paper, specifically in the central detection region covering the commercial SPE. The described paper-based devices were used for the electrochemical detection of glucose in both standard solutions and real samples (e.g., blood and soda beverages) using amperometry, DPV or cyclic voltammetry. Overall, the authors reported good analytical performances, comparable to those of conventional methodologies. Moreover, they pointed out other advantages of the devices coupling paper disks with commercial SPEs, such as the biocompatibility of the paper microenvironment, the lower volume of reagents and samples required, the separation abilities of the paper layers and the possibility to re-use the device by simply replacing the paper section [11,29,32,37].

In a different approach, Nantaphol et al. developed a cholesterol biosensor by coupling a paper analytical device with a commercial boron-doped diamond electrode [41]. The paper section had carbon counter and Ag/AgCl reference electrodes screen-printed on its hydrophilic areas. The back of the paper was applied onto double-sided adhesive tape (with a hole cut through the overlaying electrode area) which was then attached to the working electrode–a boron-doped diamond sheet. This electrode was modified with electrodeposited AgNPs, in order to increase the electrochemical specific surface area and to improve the conductivity and the electrocatalytic activity. To complete biosensor construction, ChOx was drop casted and dried atop of the hydrophilic zone of the paper support. The H_2_O_2_ generated by the enzymatic reaction in the presence of cholesterol was reduced at the NPs modified working electrode, generating a current signal that was measured by amperometry. The biosensor exhibited a high selectivity for cholesterol detection and an excellent performance in terms of detection limits (comparable to other previously reported biosensors) and for real sample analysis (bovine serum) [41].

#### 2.4.5. Alternative Procedures

Wu et al. developed a low-cost electrochemical immunosensor to detect neomycin in milk samples [27]. The sensor was fabricated by an alternative procedure that does not fit any of the methodologies already described. The bioelectrode was based on single-walled carbon nanotubes (SWCNT)/antibody composites deposited by dip–dry cycles on a paper support. The SWCNT and antibodies mixture was prepared in a solution with poly(sodium 4-styrenesulfonate) (PSS) which proved to be very efficient as a dispersion medium for the carbon material and as a protein stabilizer. The paper filter strips were coated with this mixture using a simple layer-by-layer method involving repeated cycles of dipping and freeze-drying the paper. The working electrode was constituted by the modified filter paper whereas conventional Ag/AgCl reference and Pt counter electrodes were used to complete the electrochemical cell. The biosensor’s response was measured by amperometry after a 5 min period of incubation in a solution containing neomycin. The method showed good sensitivity for neomycin and satisfactory recoveries from spiked milk samples [27].

## 3. Conclusions

The volume of analytical assays performed outside the central laboratory has expanded considerably over the last years. This is mainly due to the progress made in the construction of cost-effective POCTs. Most of these tests are tailored for the needs of low- and middle-income countries, according to the ASSURED criteria. Nonetheless, other key drivers come from the developed world where healthcare systems are putting pressure on the development of cheap, rapid and user-friendly tests, preferentially providing multiple results from a single sample; this enables timely decision-making, even in remote settings with no laboratory infrastructures [4,6]. The concept of e-health, i.e., the use of information and communication technologies (ICTs) to improve diagnosis, treatment, monitoring and management in healthcare is also stimulating the research and development of portable patient-monitoring devices that deliver care closer to the patient.

Paper-based diagnostics has clearly been gaining an increasing importance in this field, particularly regarding applications in clinical analysis. Among other advantages, paper is a versatile, cheap, biocompatible material that is also eco-friendly and easy to discard. Therefore, not surprisingly, this natural polymeric material was quickly employed as the solid substrate of electrochemical (bio)sensors. In fact, paper is an ideal material to produce custom tailored miniaturized electrodes that can work as disposable analytical tests. Besides providing quantitative readouts, electroanalytical systems allow detection in turbid samples and are less prone to errors than most common colorimetric assays. As proved by the latest developments presented throughout this manuscript, screen- and inkjet-printing are the preferential techniques for electrode manufacturing, although other alternative strategies have been proposed. The miniaturized printed electrodes can be easily coupled to biomolecules, thereby improving the analytical performance from the sensitivity and selectivity viewpoints; in this context, paper can be either used as a solid substrate or as a simple immobilization matrix where biological components are adsorbed.

Despite the very promising results, the research and development on paper-based electrochemical biosensors is a recent trend, and no mature platform has yet appeared. In the near future, the translation of current research prototypes into commercial applications should mainly address challenges related with quality assurance [3]. One of the most critical aspects to be tackled is the achievement of reproducible quantitative results; this depends not only on the test’s performance and the capability of producing identical sensor batches but also on the environmental variables, which can affect the operating conditions and consequently, the signal output [8]. As such, POCTs are often more at risk of interferences than conventional laboratory assays [6]. A second aspect relates with the difficulty on developing simple-to-use tests for non-laboratory trained individuals [8], who are more prone to making mistakes leading to deviations in measured results than competent staff in well-run laboratories. Last but not the least, the final cost of the commercial product could be another obstacle to surpass [6]; for this reason, a great hope has been placed on paper-based POCTs.

## Figures and Tables

**Table 1:** Characteristics of paper-based electrochemical biosensors.

Reference	Paper	Hydrophobic Patterning	Working Electrode	Electrode Modification Materials	Biorecognition Element	Analyte
Dungchai 2009 [24]	Whatman #1	Photolitography	SPCE array	Prussian Blue	GOx, Lactate Oxidase, Uricase	Glucose, Lactate, Uric Acid
Nie 2010 [23]	Whatman #1	Photolitography or wax printing	SPCE	Ferricyanide	GOx	Glucose and Pb(II)
Jagadeesan 2012 [25]	Whatman #1	–	SPCE	PANI, ferricyanide in solution	Antibodies	Troponin
Tan 2012 [26]	Whatman #1	–	Commercial SPCE	–	GOx	Silver ions
Wu 2012 [27]	Filter paper strips	–	SWCNT	–	Antibodies	Neomycin
Määttänen 2013 [28]	Multilayer-coated recyclable paper	PDMS ink	Gold SPE	PEDOT	GOx	Glucose
Noiphung 2013 [29]	Whatman #1 and VF separation paper	Wax dipping	Commercial Prussian Blue SPCE	Prussian Blue	GOx	Glucose
Santhiago 2013 [30]	Whatman #1	Wax printing	Graphite-pencil	4-aminophenylboronic acid	GOx	Glucose
Zhao 2013 [31]	Whatman #1	Wax printing	SPCE array	Ferricyanide	GOx, Lactate Oxidase, Uricase	Glucose, Lactate, Uric Acid
Kong 2014 [32]	Whatman #1	–	Commercial SPCE	Graphene, PANI, AuNPs	GOx	Glucose
Labroo 2014 [33]	Regular paper	Wax printing	Inkject printed graphene	–	GOx, Lactate Oxidase, XO, ChOx	Glucose, Lactate, Xanthine, Cholesterol
Lawrence 2014 [11]	Whatman #1	–	Commercial SPCE	Ferrocene monocarboxylic acid	GOx	Glucose
Li 2014 [34]	Whatman #1	Wax printing	SPCE	AuNPs, MnO_2_ nanowires	Antibodies	PSA
Ruecha 2014 [35]	Whatman #1	Wax printing	SPCE	Graphene, polyvinylpyrrolidone and PANI	ChOx	Cholesterol
Wu 2014 [36]	Whatman #1	SU-8 photoresist	SPCE array	Graphene oxide, chitosan and glutaraldehyde	Antibodies	Cancer biomarkers
Yang 2014 [37]	Whatman #1	–	Commercial SPCE	AgNPs	GOx	Glucose
Ge 2015 [38]	Chromatographic	Wax printing	SPCE	AuNPs, graphene, IL	Concanavalin A	K-562 cells
Ge 2015 [39]	Chromatographic	Wax printing	SPCE	AuNPs	Folic acid	K-562 cells
Li 2015 [40]	Whatman #1	Wax printing	SPCE	–	Labeled DNA probe	viral DNA
Nantaphol 2015 [41]	Whatman #1	Wax printing	Boron-doped diamond	AgNPs	ChOx	Cholesterol
Wu 2015 [42]	Whatman #1	Wax printing	SPCE	3-aminopropyldimethoxysiloxane, NAD^+^ and ferricyanide	ADH	Ethanol
Fischer 2016 [43]	Whatman #1	Wax printing	SPCE	Chitosan	GOx	Glucose
Li 2016 [44]	Whatman #1	Not specified	Pencil drawn graphitic layers	Ferrocenecarboxylic acid	GOx	Glucose

ADH–alcohol dehydrogenase; AgNPs–silver nanoparticles; AuNPs–gold nanoparticles; ChOx–cholesterol oxidase; GOx–glucose oxidase; IL–ionic liquid; NAD^+^–nicotinamide adenine dinucleotide; PANI–polyaniline; PEDOT–poly-3,4-ethylenedioxythiophene; PDMS–polydimethysiloxane; PSA–prostate protein antigen; SPCE–screen-printed carbon electrode; SPE–screen-printed electrode; ssDNA–single-stranded DNA; SWCNT–single-walled carbon nanotubes; XO–xanthine oxidase.
